# A novel method in myocardial injury risk stratification using intravenous fat emulsion as sole rapid preparation for unfasted patients to suppress myocardial 18F-fluorodeoxyglucose uptake for optimal cardiac PET imaging: a proof-of-concept randomized-crossover trial

**DOI:** 10.3389/fnume.2024.1412917

**Published:** 2024-10-23

**Authors:** Michael H-G. Li, Raef R. Boktor, Christopher Rowe, Laurence Weinberg, Bernhard Riedel

**Affiliations:** ^1^ Department of Anaesthesia, Austin Health, Heidelberg, VIC, Australia; ^2^ Department of Anaesthesia, Perioperative and Pain Medicine, Peter MacCallum Cancer Centre, Melbourne, VIC, Australia; ^3^ Department of Molecular Imaging and Therapy, Austin Health, Heidelberg, VIC, Australia; ^4^ Olivia Newton-John Cancer Research Institute, Heidelberg, VIC, Australia; ^5^ School of Cancer Medicine, La Trobe University, Bundoora, VIC, Australia; ^6^ Florey Department of Neuroscience and Mental Health, Austin Health, Heidelberg, VIC, Australia; ^7^ Department of Critical Care, The University of Melbourne, Parkville, VIC, Australia; ^8^ The Sir Peter MacCallum Department of Oncology, The University of Melbourne, Parkville, VIC, Australia

**Keywords:** cardiac imaging techniques, fat emulsion, intravenous, positron emission tomography computed tomography, PET, 18F-fluorodeoxyglucose, ^18^F-FDG, healthy volunteers

## Abstract

**Objectives:**

Optimal imaging of ischemic or inflammed myocardium via ^18^F-FDG PET imaging requires suppression of background carbohydrate metabolism in normal myocardium. Sole administration of intravenous lipid emulsion has not previously been used to rapidly prepare unfasted patients, such as in emergent clinical situations. In this proof-of-concept pilot, we posited that intravenous fat emulsion suppresses physiological metabolic uptake of in non-ischemic, non-inflammatory myocardium in unprepared and unfasted setting for enhanced cardiac positron emission tomography (PET) imaging.

**Methods:**

We conducted an ethics-approved, single-blind, prospective randomized crossover trial of 10 healthy volunteers from January 2020 to June 2021. Participants were unfasted and rendered hyperglycemic before being administered either high dose intravenous lipid emulsion—1.5 ml kg of 20% lipid emulsion, followed by 15 ml/kg/hr for 30mins—or saline prior to ^18^F-FDG injection and subsequent cardiac PET imaging. Assessors undertook image analysis for maximum standard uptake value (SUVmax), minimum standard uptake value (SUVmin) and qualitative assessment, and groups were compared using univariate analysis.

**Results:**

The study population age was 44.5 years [IQR 32.5–56.5], with 50% male and a median BMI of 22.75 [IQR 25.0–28.5] kg/m^2^. The study was feasible and there were no adverse side effects from the interventions. In these participants with normal myocardium, ^18^F-FDG uptake was reduced by intravenous lipid emulsion as assessed by SUVmax and qualitative assessment (*p* = 0.042, *r* = 0.454 and *p* = 0.009, *r* = –0.581, respectively).

**Conclusions:**

Intravenous lipid emulsion suppresses background metabolic uptake of ^18^F-FDG even in unprepared and unfasted patients. Our findings prove and expand the possible applications for cardiac ^18^F-FDG PET in various settings, including in emergent settings as a means of rapid preparation in place of current more time-consuming standard protocols, allowing time-critical management to be effected.

## Introduction

Postoperative myocardial injury is an important and clinical relevant diagnosis due to significantly increased risk of mortality and major vascular complications 30 days to 2 years post-surgery (1) To that end, it is unsurprising that the search for a viable diagnostic tool of myocardial injury and risk stratification continues.

In recent years, the availability and clinical efficacy of positron emission tomography (PET) has grown such that it has developed from a research technique into a viable clinical tool for the diagnosis of various heart diseases (2). Imaging using 18F-fluorodeoxyglucose (^18^F-FDG) allows for direct assessment of myocardial metabolism and therefore can, with appropriate preparation, can determine the presence or absence of regional ischemia or inflammation (3). This is possible due to the physiologic characteristics of normal myocardial metabolism, where profound changes in substrate utilization occur with the onset of myocardial ischemia. During conditions with normal myocardial perfusion, particularly in the fasted state and after a high-fat, low-carbohydrate meal (4), the preferred myocardial energy substrate is free fatty acids (5–7). In the presence of inflammation, ischemia, and consequent anerobic conditions, myocardial metabolism shifts the preferred energy substrate from free fatty acids to glucose (8). This is demonstrated via PET imaging as increased ^18^F-FDG radiotracer uptake in acutely ischemic or inflamed myocardium (9).

There is currently a need in the field of risk stratification of myocardial injury after non-cardiac surgery, prosthetic valve endocarditis and inflammatory disorders such as cardiac sarcoidosis that FDG PET may be able to fill (10, 11). Optimal imaging, however, requires suppression of background carbohydrate metabolism (and FDG uptake) in normal non-ischemic, non-inflamed myocardium. Current protocols that exist for elective cardiac PET imaging, e.g., for cardiac sarcoidosis (12), use strategies such as a low-dose heparin infusion or a prolonged fasting or a very high-fat, low-carbohydrate, protein-permitted diet (13). These protocols are not compatible with emergent care such as imaging for acute myocardial ischemia, and on their own.

The currently accepted PET preparation protocol stipulates that patients fast for between 4 and 12 h before scanning. In practice, patients are usually advised to fast for at least six hours before a PET scan but to drink at least 500 ml of plain water in the two hours before scanning. However, variable physiologic uptake of myocardial ^18^F-FDG using this standardized fasting protocol has been shown to yield false-positives, resulting in difficulties in the interpretation of the results of these PET studies (14, 15). The very high-fat, low-carbohydrate, protein-permitted (VHFLCPP) diet preparation protocol was introduced based on the Randle effect (16), a competitive metabolic process between glucose and free fatty acids as the preferred myocardial energy substrate. While the mechanism of inhibition of glucose uptake is not yet entirely understood, it is thought that GLUT-4 channel upregulation, the rate-limiting step in the myocardium, and therefore insulin dependent, may play a role (17). With the myocardial tissues preferentially metabolizing free fatty acids, it appears that a VHFLCPP diet protocol reduces myocardial ^18^F-FDG uptake after the post-prandial hyperinsulinemic peak period (16). Conversely, in myocardial ischemia, glycolysis becomes the predominant source of energy production. The inefficiency of glycolysis, coupled with myocardial ischemia, results in the translocation of GLUT-4 and GLUT-1 from the sarcoplasm to the sarcolemma (3).

Intravenous lipid emulsion, in conjunction with heparin, both via action on the GLUT-4 channel, has been shown to reduce both insulin and plasma glucose uptake in human myocardium under a euglycemic clamp technique (18). Therefore, we hypothesized that increasing plasma free fatty acids via intravenous lipid emulsion infusion in a hyperglycemic state, to replicate the emergent referral for imaging in an unfasted state, would reduce myocardial glucose uptake in normal non-ischemic, non-inflamed myocardium.

## Methods

We conducted a feasibility study via a prospective single-blinded, randomized-controlled crossover trial among 10 healthy volunteers aged 18 years or older in an outpatient setting between January 2020 and June 2021. Exclusion criteria included prior diagnoses of diabetes mellitus, ischemic heart disease, cardiac surgery, body mass index (BMI) less than 18 or greater than 30 kg/m^2^, allergies to lipids, glucose, soya bean, peanut or egg, pregnancy, or claustrophobia. Following prospective trial registration (Australian & New Zealand Clinical Trials Registry, ACTRN12616000279426p) and ethics approval (HREC/18/Austin/203), participants were recruited via convenience sampling, medically screened, then randomized with cross-over to both the intervention and control arms in a 1:1 ratio. There was a minimum period of 1 week between the two study arms for each volunteer. Following a cross-over design, patients who received the intervention subsequently received the control design and patients who received the control first waited at least a week before receiving the intervention.

Participants were unfasted regardless of whether they were to receive intravenous lipid emulsion or saline and were rendered hyperglycemic to a blood glucose level greater than 10 mmol/L with oral, followed by intravenous glucose that was administered before the study drug (intravenous lipid emulsion or saline). This was to simulate the conditions of patients being unprepared and therefore unfasted in emergent settings where adequately fasting a patient 4–6 h prior to cardiac PET scans were not possible.

To ensure participants were unfasted, even hyperglycemic, a 25 g oral glucose load was first administered, with blood glucose level checked at 15 min. Further administration of 10 ml 50% dextrose was then administered with checks every 5 min until the blood glucose level reached greater than 10 mmol/L.

Either intravenous lipid emulsion [Intralipid^TM^ (Frasenius Kabi, Bad Homburg vor der Höhe, Germany)] or saline were administered with a 1.5 ml/kg bolus followed by an infusion of 15 ml/kg/h for 30 min. Immediately thereafter, the subjects received intravenous injection of ^18^F-FDG [100MBq (3 mCi)] and were kept in a dim, relaxing room for 60 min uptake time before scanning.

Thereafter, non-gated with ultra-low attenuation CT acquisition via Philips TF64 PET computed tomography (CT) scanner was performed. Acquisition was limited to a single bed step with the heart in the center, for a total acquisition time of 10–20 min. Participants had their vital signs recorded and were monitored for adverse effects up to 30 min following the CT scan.

The primary study outcomes were feasibility measures. Feasibility outcomes were defined as the ability to achieve a blood glucose level of greater than 10 mmol/L with either oral glucose or intravenous dextrose in all participants, and the successful drug administration of high-dose intravenous lipid emulsion to all participants. Secondary outcomes were the assessment of safety of intravenous lipid emulsion and injection of ^18^F-FDG [100MBq (3 mCi)]. The key efficacy outcome was whether intravenous lipid emulsion reduced normal myocardial ^18^F-FDG uptake.

The reconstructed attenuation-corrected PET images were assessed by two blinded, specialist nuclear medicine physicians (RB, CR), with greater than 20 years’ experience in the relevant reporting techniques, to determine image quality and produce clinical interpretations. A qualitative analysis of myocardial ^18^F-FDG uptake was performed of myocardial ^18^F-FDG uptake. The blinded reader used a qualitative visual categoric uptake scale to assess the ^18^F-FDG uptake by the myocardium visually (homogeneously minimal, or less than the blood pool—score of 0; mild, patchy diffuse uptake 1; mild, homogenous uptake, more than blood pool—score of 2; moderate, patchy diffuse uptake—score of 3; moderate, homogenous uptake, less than liver uptake—score of 4; homogeneously intense, or uptake greater than liver—score of 5) (19). Blood pool was considered to be the activity within the heart cavity or large vessels (e.g., aorta). Semi-quantitative analysis determined the minimum standardized uptake value (SUV_min_) and maximum SUV value (SUV_max_) in each subject's myocardium. The standardized uptake value (SUV) is a semi-quantitative measure of the uptake in the region of interest. It normalizes the activity in the region of interest to the injected activity and is a measure of volume distribution. A region of interest was drawn on the myocardium and the lowest value was determined to be SUV_min_ and the highest was determined to be SUV_max_.

### Randomization

Participants underwent simple randomization using a computer-generated program. Ten opaque envelopes were prepared by an independent person and marked Participant 1, up to Participant 10A, with each envelope containing details of the the initial arm (intervention or control). Envelopes were held by independent personnel until the day of intervention.

### Statistical analysis

This was a proof-of-concept study to demonstrate feasibility and generate data to inform the design and power a subsequent definitive clinical trial. Therefore, a convenience sample of ten subjects were enrolled. Baseline patient characteristics were summarized using descriptive statistics and were reported for continuous variables as the number of patients, mean, standard deviation, median, inter-quartile range, minimum and maximum, depending on data distribution. Categorical variables were reported as counts and percentages. All patients who completed the ^18^F-FDG PET study were included in the feasibility analysis. Endpoints of quantitative SUV measurements and the semi-quantitative visual scale for cardiac ^18^F-FDG PET imaging quality were compared between groups with a Wilcoxon signed rank at *α* = 0.05. We reported the study using the Consolidated Standards of Reporting Trials (CONSORT) guidelines (20).

## Results

Of 13 screened (2 refused due to concerns regarding radiation exposure, and 1 regarding insufficient time commitment), 10 patients were successfully enrolled in the study. The median study population was 44.5 years [IQR 32.5–56.5], with 50% male and a median BMI of 22.75 [IQR 25.0–28.5]. (Table 1) No patients reported undertaking the VHFLCPP diet before the study. Intravenous lipid emulsion and ^18^F-FDG [100MBq (3 mCi)] were successfully administered to all participants, and all participants were analysed according to their original allocated groups. Of the 10 randomized participants, 5 to the saline first arm, and 5 to the fat emulsion arm, all of the participants were able to complete the study. For three of the 20 scans, two saline cases and one intravenous lipid emulsion case the study participants were fasted for six hours before induction of hyperglycemia. All cases required intravenous dextrose to achieve a blood glucose level of greater than 10 mmol/L. There were no statistically significant differences between pre-infusion blood glucose levels (*p* = 0.153) or post-infusion blood glucose levels (*p* = 0.507) between the intravenous lipid emulsion and saline cases. Minor adverse events were experienced in some participants that received intravenous lipid emulsion, with three participants having a metallic taste and two having self-resolving superficial thrombophlebitis. Ultimately, all infusions were administered to their total dose and were well tolerated.

**Table 1:** Demographics.

Demographics	*n*=10
Age, median [IQR]	44.5 years [32.5–56.5]
Gender (male), %	50
Height (cm), median [IQR]	177.5 [162.00–182.0]
Weight (kg), median [IQR]	75.5 [70.3–80.5]
BMI, median [IQR]	22.8 [IQR 25.0–28.5]
Ethnicity	
Caucasian	5
Non-Caucasian	5

Baseline characteristics of all enrolled participants.

**Figure 1:**
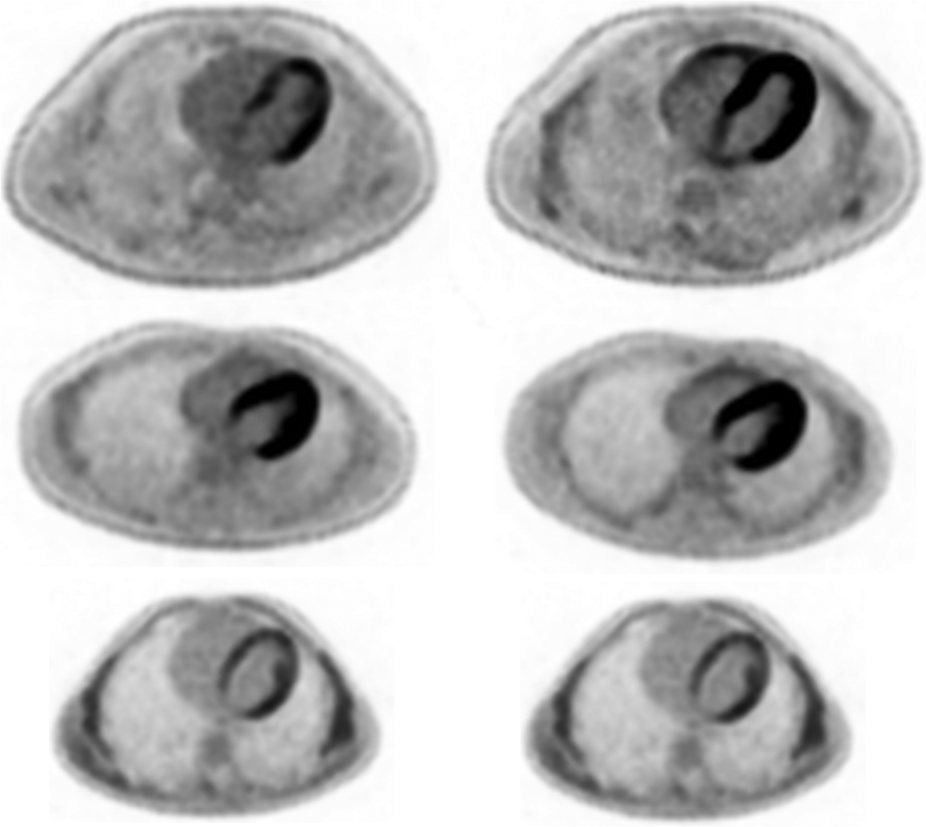
Comparative images between fat emulsion (left) and saline (right) for cases 2 (first row), 6 (second row) and 10 (third row), with case 2 demonstrating a larger difference in SUVmax compared to cases 6 and 10. Case 6 showed a relatively high SUVmax compared to case 10, despite both showing relatively similar uptake between groups.

Regarding our main efficacy outcome (Table 2), ^18^F-FDG uptake was statistically reduced by intravenous lipid emulsion as assessed by SUVmax and qualitative assessment (*p* = 0.009, *r* = 0.454 and *p* = 0.042, *r* = –0.581, respectively). There were no statistically significant differences between SUVmin assessments. Variability in the data, including extremes in the uptake in select cases, is illustrated in Figure 1. Rib uptake confirmed artificially-induced hyperglycemia, although this was highly variable. There were no correlations between the SUV_max_ and the pre-infusion blood glucose level (*p* = 0.453) or post-infusion blood glucose level (*p* = 0.789). Individual results are summarized in Table 3.

**Table 2:** Semi-quantitative and qualitative analysis of intervention and control groups.

	Intravenous lipid emulsion	Saline	*p*-value
SUVmin, median [IQR]	0.205 [0.16–0.26]	0.23 [0.16–0.27]	*p* = 0.857
SUV_max_, median [IQR]	4.05 [2.75–5.74]	6.81 [3.62–8.33]	*p* = 0.009
Qualitative, median [IQR]	4.00 [1.75–5.00]	5.00 [4.00–5.00]	*p* = 0.042

Comparison of semi-quantitative and qualitative ^18^F-FDG PET image analysis in participant groups administered intravenous lipid emulsion or saline. SUV, standardized uptake value; Qualitative scoring: 0, no uptake; 1, mild, patchy uptake; 2, mild, diffuse uptake; 3, moderate, patchy uptake; 4, moderate diffuse uptake; 5, homogenously intense.

**Table 3:** Individual cases.

Case	Intralipid			Saline		
	SUV Min	SUV Max	Qualitative	SUV Min	SUV Max	Qualitative
1	0.29	3.49	5	0.33	3.9	5
2	0.16	4.6	4	0.2	11.7	5
3	0.25	2.68	2	0.18	8.98	5
4	0.19	6.79	5	0.25	8.11	5
5	0.15	4.49	3	0.11	6.58	5
6	0.22	8	5	0.23	7.95	5
7	0.12	3.61	2	0.08	4.64	3
8	0.17	5.39	5	0.23	7.04	5
9	0.38	1.74	1	0.31	2.16	3
10	0.23	2.77	3	0.23	2.78	3

Comparison of semi-quantitative and qualitative numbers.

## Discussion

No prior studies have examined the use of intravenous fat emulsion alone in image optimization for ^18^F-FDG cardiac PET imaging. Our study successfully provided proof-of-concept that intravenous lipid emulsion can variably reduce myocardial ^18^F-FDG uptake in healthy volunteers without severe adverse effects. Although it does not provide full suppression, intravenous lipid emulsion improves PET image quality in unfasted and/or hyperglycemic patients by reducing both semi-quantitative SUV_max_ and qualitative ^18^F-FDG uptake. It does so without severe side effects and is shown to be well-tolerated, suggesting there is a role for intravenous fat emulsion to play; if not as a primary induction agent for myocardial suppression in cardiac PET imaging, then certainly as a highly useful adjunct to image optimization.

As a viable adjunct, future applications of intravenous fat emulsions may include being used in ^18^F-FDG cardiac PET imaging when confirming and quantifying cardiac ischemia, inflammation and infection in both emergent and non-emergent settings, or when adherence to dietary preparation is suboptimal.

### Fat emulsion as an additional adjunct to other techniques

No prior studies have examined the use of intravenous fat emulsion alone in image optimization for ^18^F-FDG cardiac PET imaging. Recently, in relatively low doses, it has been shown to be effective as an adjunct to improve image quality in patients undergoing a VHFLCPP diet (21). As study participant preparation was intentionally suboptimal in our study, it is difficult to comment on the dose-related myocardial reduction, although our study suggests that much higher doses of intravenous fat emulsion would be tolerated, and could potentially contribute to image optimization, but such benefits likely have a “ceiling effect”. Additionally, heparin has been shown to improve reduction in approximately one in 10 patients following the same diet (22). The proposed mechanism of action of heparin in the myocardium is not entirely clear, but it has been shown that heparin reduces GLUT-4 translocation in skeletal muscle by interacting with insulin and inhibiting the insulin-mediated activation of the PI3K/Akt signalling pathway (23).

In an ideal situation, each intervention (VHFLCPP, fat emulsion, and heparin) appears to provide an additive component of myocardial reduction. However, in semi-urgent situations, fasting or a VHFLCPP diet may not be practicable. In post-surgical situations, the use of heparin at doses required for myocardial reduction (15–50 units/kg) may not be appropriate. Fat emulsion, therefore, is a good alternative to these situations. Whilst there appears to be a degree of individual variability in response to fat emulsion and ^18^F-FDG uptake, a larger study would be able to determine to what degree this variability exists.

### Utility of high-dose intravenous fat emulsion in inflammatory cardiac disease

The use of fat emulsion for inflammatory cardiac disease has been variably examined. Recently, it has been implicated for its potential use in diagnosing endocarditis, particularly of prosthetic valves (24, 25), where delays in imaging can lead to negative confounding due to the effects of low inflammatory activity from prolonged antibiotic therapy (26). Using a VHFLCPP diet preparation, Saby et al. has shown that PET/CT may have a significant role in improving the sensitivity of the Duke criteria of prosthetic valve endocarditis from 73% to 93% (11). In the context of our results and additional literature, we hypothesize that the addition of fat emulsion will further improve imaging quality, particularly in emergent cases which would benefit from urgent surgery. In such cases, we demonstrate that a high-dose fat emulsion is may be a potential adjunct to additional image optimization strategies. PET/CT also has the additional benefit of identifying septic emboli, which provides significant prognostic implications (27). It is worth noting that a systemic hypercoagulable state, while not demonstrated in our study, would increase the risk of thromboembolism in prosthetic valve endocarditis (28). In cardiac sarcoidosis, current guidelines include a VHFLCPP diet, followed by fasting, and low-dose intravenous heparin (29). No studies have examined the use of intravenous fat emulsion in a non-emergent setting.

### High-dose intravenous fat emulsion in other settings

Intravenous fat emulsion is not without its risks, with including an early, but low risk of hypercoagulability and irritation, which are mild and transient and is thought to be less than 1% in incidence (30). While it did not affect the conduct of this pilot study, these effects were increased in our small sample size, which may be due to the higher dose range or may possibly a spurious result. As discussed above, a larger study could further elucidate us, and the effects can be better examined in future post-recovery studies. Given that there is no established link between superficial thrombophlebitis and a systemic hypercoagulable state (31), it is more likely that this side effect was directly-related to local irritation at the site of injection.

More importantly, our study adds to limited literature of high-dose intravenous emulsion administration. No study to date has examined the use of high dose intravenous fat emulsion in an elective setting or in otherwise healthy adult patients, with the majority of literature limited to case reports of its use as rescue therapy in critically ill patients from drug overdose, for example, local anesthetic toxicity (32, 33). Of these reports, only two cases describe asystolic cardiac arrest following fat emulsion administration, but other factors make it difficult to determine causality (34). Recommendations suggest a maximum dose of 10 ml/kg of 20% intravenous fat emulsion (35), but animal studies have suggested a dose of 60 ml/kg is considered safe (36). Until now, no data has been published to justify a maximum dose in humans.

### Limitations and strengths

In addition to a small convenience sample of ten subjects, which is common in many proof-of-concepts studies, our study has a number of limitations. We did not measure the intravenous free fatty acid levels and were, therefore, unable to determine the maximal effect of the drug before ^18^F-FDG injection. There were some extreme variations between uptake in some subjects. As we did not standardize the subjects’ diet before induction of hyperglycemia, it is also unclear whether differences in diet may have accounted for some individual variation. There are potentially other unaccounted for metabolic factors that could also account of some of the variability. Participants were typically younger, thus the degree of myocardial glucose uptake, and therefore suppression, may be altered in the relatively elderly population (37). We also excluded patients with coronary artery disease and diabetes, which has issues with respect to applicability in these populations. Despite this, our study has several strengths. First, we used a relatively larger dose of intravenous fat emulsion than other studies over a shorter period. Therefore, the results of our study are unlikely to be due to inadequate dosing and rules out the need to trial higher doses. Second, we also provided the least optimal conditions for cardiac imaging by rendering the participants profoundly hyperglycemic in the 30 to 45-minute period prior to ^18^F-FDG injection, and the image quality will likely be improved in a clinical situation. Finally, our study did not have any severe or life-threatening adverse reactions to a rescue dose of intravenous fat emulsion in healthy patients, which has implications for its use and study in other emergent settings as a rescue drug.

## Conclusion

Our study suggests that intravenous fat emulsion can variably reduce myocardial ^18^F-FDG uptake in unfasted, healthy volunteers. This poses some possibilities in its role as an adjunct during emergent and semi-emergent settings, as well as building upon the safety effect profile of high-dose intravenous fat emulsion. As such, further investigation into how this approach, in conjunction with methods such as heparin administration, is warranted if we hope to fully develop a practical ^18^F-FDG scanning protocol, especially for use in settings as discussed, including studies to improve image evaluation for diagnostic accuracy and to better clinically relevant interpretation for patients with known cardiac pathology in semi- and acute settings.

## Data Availability

The raw data supporting the conclusions of this article will be made available by the authors, without undue reservation.
